# Chronic kidney disease and itch

**DOI:** 10.1097/itx.0000000000000076

**Published:** 2024-09-20

**Authors:** Seyyede Zeinab Azimi, Ethan A. Lerner

**Affiliations:** Center for Research & Training in Skin Diseases & Leprosy, Tehran University of Medical Sciences, Tehran, Iran and Department of Dermatology, Cutaneous Biology Research Center, Massachusetts General Hospital and Harvard Medical School, Boston, MA

**Keywords:** Chronic kidney disease, Pruritus, Difelikefalin, Itch, Mas-related G protein–coupled receptors

## Abstract

Chronic kidney disease–associated pruritus (CKD-aP) is a prevalent and challenging symptom in patients with CKD and end-stage renal disease (ESRD). The aim of this review is to update existing evidence on the pathogenesis and treatments of pruritus in CKD and to shed light on areas that hold promise. The uncertain pathogenesis, and thus seemingly miscellaneous causes, identifies chronic itch as an important challenge in health care. A complex interaction of uremic toxin accumulation, micro and systemic inflammation, dysregulation of the opioid system, and mast cell activation may each contribute to the pathophysiology of CKD-aP. No highly satisfactory antipruritic therapeutics are available. Difelikefalin, considered to be a peripherally acting highly selective kappa-opioid receptor agonist, has been shown to have a positive impact on CKD-aP. Approved by the FDA in 2021 for intravenous administration, difelikefalin remains the most recent drug available. A developing area is that altered hemoglobin metabolism may lead to the activation of mas-related G protein–coupled receptors (MRGPRs). As this family of receptors is associated with itch, it is possible that drugs that target certain MRGPRs may be of future benefit in CKD-aP.

## Introduction

Patients with chronic kidney disease (CKD), especially such patients on hemodialysis (HD) and thus having limited kidney function, are at an increased risk of pruritus. CKD-associated pruritus (CKD-aP), previously called uremic pruritus, negatively affects the quality of life, including sleep^[1–8]^. Here, we discuss the prevalence, etiology, and treatment of CKD-aP. We aim to emphasize an evidenced-based understanding of pathogenesis and management to prompt a thought-provoking narrative for the understanding and treatment of this challenging condition^[9]^.

## Epidemiology

A meta-analysis showed that the prevalence of CKD-aP ranges from 18% to 97.8% among 42 cross-sectional studies, and the overall prevalence of CKD-aP in patients with end-stage renal disease was 55% (95% CI, 49–61)^[10]^. The largest international cohort study assessing CKD-aP prevalence in HD patients was the Dialysis Outcomes and Practice Patterns Study (DOPPS). This study investigated CKD-aP in 18,801 HD patients in 12 different countries. The prevalence of moderate-to-severe itch was 45% in DOPPS I (1996–2001) and 42% in DOPPS II (2002–2004). The results confirmed a decreasing trend in CKD-aP prevalence^[11]^. The follow-up assessment study of DOPPS IV-VI (2009–2018) revealed that the prevalence of moderate-to-severe itch decreased to 37%^[2,4]^. A similar cohort study in Japan that examined dialysis outcomes and practice patterns between 1999 and 2004 (JDOPPS) revealed that moderate-to-extreme pruritus was 44% in HD patients^[12]^.

Duque et al^[13]^ evaluated 105 HD patients in a cross-sectional study and reported that the prevalence of CKD-aP was 57.1%. In another cross-sectional study in Iran, 237 HD patients were asses sed for CKD-aP, and the prevalence of CKD-aP was 54.08%^[14]^. The highest prevalence was observed in China, where CKD-aP was reported in 97.8% of 182 HD patients^[15]^. Also, a high prevalence of 85.4% of CKD-aP was reported from a cross-sectional study in Turkey^[16]^. A cross-sectional study investigated 860 German HD patients and revealed a CKD-aP prevalence of 18% among HD patients^[17]^.

Differences in the prevalence among studies could be due to definitions, inclusion criteria, and assessment methods used for CKD-aP diagnosis as well as regional, racial, and economic differences^[10,18]^. Rayner et al^[2]^ highlighted how strongly nephrologists underestimated CKD-aP. They found that 65% of medical directors expected fewer than 5% of their patients to have severe pruritus, 17% of HD patients with CKD-aP did not express their symptoms, and 18% did not receive any therapy. In a cross-sectional survey, Weisshaar and colleagues showed that most of the nephrologists (204 responders) predicted that fewer than 30% of HD patients had CKD-aP. The authors proposed that their observation might be due to the absence of effective treatment for CKD-aP^[19]^. Patient and clinician attitudes concerning the importance of itch as a health problem could be a factor that influences whether or not itch is reported. Accepting pruritus as something to live with, lack of interest, and prioritizing other health concerns among patients with CKD, as well as lack of attention of clinicians for itch assessment through consultations, could also contribute to the discrepancies^[20]^.

## Pathogenesis

**Figure 1.**
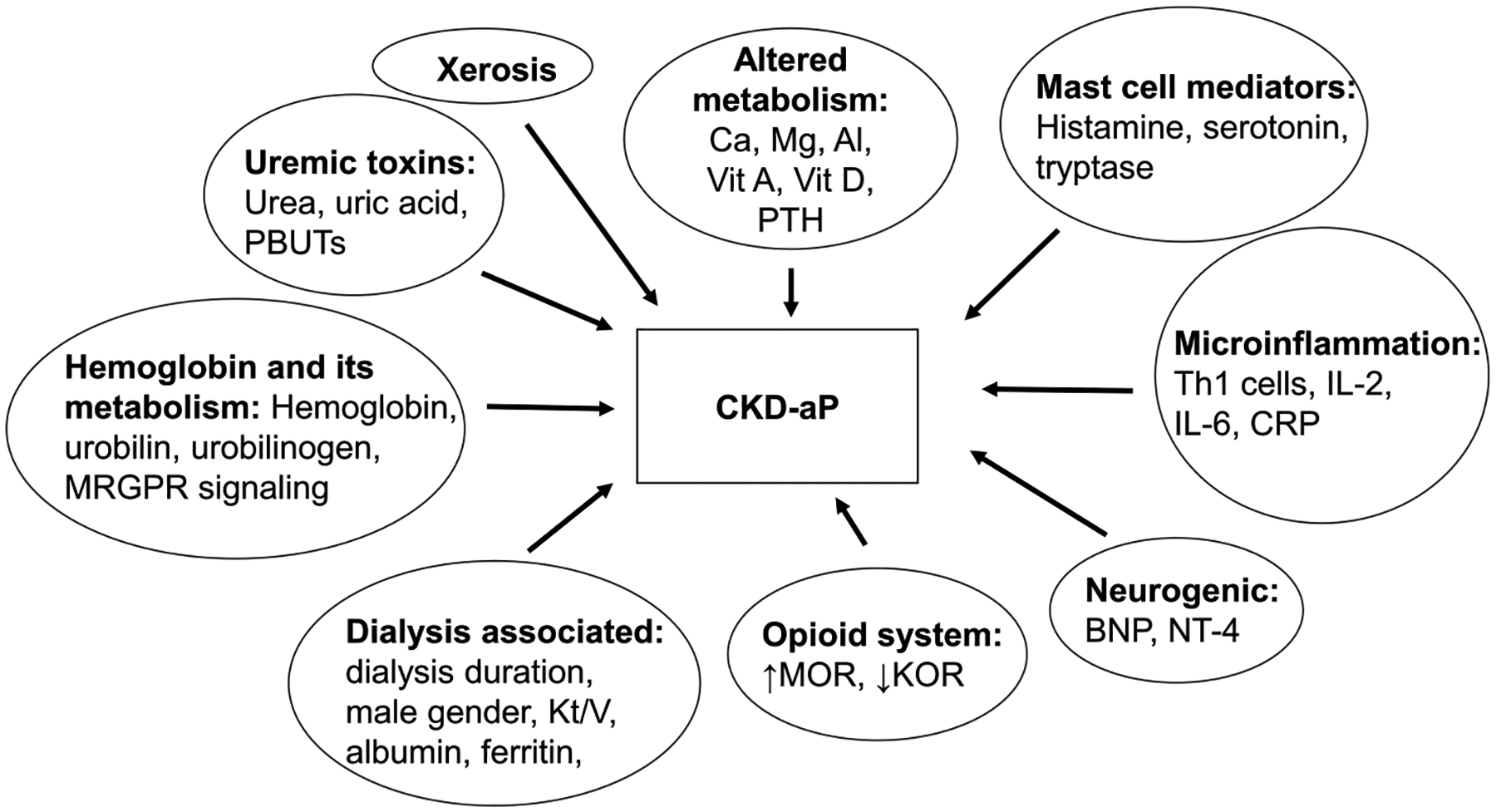
Pathophysiology of chronic kidney disease–associated pruritus. Al indicates aluminum; BNP, brain natriuretic peptide; Ca, calcium; CRP, C-reactive protein; IL-2, interleukin 2; IL-6, interleukin 6; KOR, ƙ-opioid receptor; Mg, magnesium; MOR, μ-opioid receptor; MRGPR, Mas-related G protein–coupled receptors; NT-4, Neurotrophin-4; PBUTs, protein-bound uremic toxins; PTH, parathyroid hormone; Th-1, T helper 1; WBC: white blood cell.

The pathophysiology of the chronic itch is multifaceted and understood partially^[21]^. It is thought that keratinocytes, immune cells, and adjacent neurons produce substances, including histamine, prostaglandins, cytokines, neuropeptides, and proteases, which can trigger itch^[22,23]^. These pruritogens stimulate G protein–coupled receptors, Toll-like receptors, or interleukin receptors on primary afferent sensory neurons whose cell bodies are in the dorsal root and trigeminal ganglia. These primary afferents synapse with second-order interneurons of the dorsal horn of the spinal cord. Spinal interneurons communicate with projection neurons in the cerebral cortex^[24]^. Potential con tributors to the pathophysiology of CKD-aP are depicted schematically in Figure 1.

### Uremic toxins

A longstanding hypothesis has been that uremic toxins accumulate in the skin and subcutaneous tissue and act as possible pruritogens. In CKD, inappropriate removal of metabolite substances results in their accumulation in the body. These compounds, called uremic toxins, can be categorized into 3 subgroups: small (< 500 Da) water-soluble solutes (eg, urea), medium-sized molecules, and protein-bound toxins. During dialysis, low–molecular weight molecules are easily removed. Uric acid is one of the small water-soluble solutes that impacts the progression of CKD and associated mortality risk. The impact of uric acid on CKD-aP is controversial^[25]^. Wang et al^[26]^ showed that pruritus was associated with higher levels of uric acid among 320 patients with CKD. Other studies reported contrary findings^[27,28]^. Protein-bound uremic toxins (PBUTs) [eg, indoxyl sulfate (IS) and p-cresyl sulfate (PCS)] are mainly excreted by renal tubular secretion and poorly removed by conventional dia lysis because of their strong protein-binding. Increasing evidence suggests that the accumulation of PBUTs may contribute to an increased risk of pruritus. Wang and colleagues could also show that patients with CKD-aP had higher total levels of IS and PCS than patients without the itch. Total PCS levels were significantly associated with pruritus severity^[26]^. Kim et al^[29]^ showed that uremic solutes such as PBUTs either directly or indirectly upregulate protease activator receptor-2 (PAR2) expression in the epidermal keratinocytes samples of CKD subjects and could have a potentially important role in the CKD-aP pruritus. Also, Moon et al^[30]^ found elevated epidermal expression of PAR2 as well as a positive correlation between PAR2 expression and itch severity score in ESRD patients. Given that PAR2 acts in a histamine-independent pruritic pathway, the possible role of this receptor in CKD-aP etiopathogenesis should be considered^[31]^.

### Altered metabolism in CKD

Metabolite imbalances of calcium, phosphorus, aluminum, magnesium, vitamin D, and vitamin A occur in CKD^[24,31]^. Blachley et al^[32]^ found an increased number of divalent ions in CKD-aP skin biopsies and suggested that microprecipitation of calcium or magnesium phosphate can cause pruritus. Momose et al^[33]^ found that the epidermis of patients with moderate-to-severe itch contained higher deposition of calcium ions in the basal layer compared with patients without pruritus. Parathyroid hormone (PTH) has been suggested as a pruritogenic substance by some studies. In CKD, hyperphosphatemia, hypo calcemia, and decreased calcitriol production can result in elevation of PTH production, and subsequently, secondary hyperparathyroidism occur^[34]^. Parathyroidectomy has been reported to result in the alleviation of CKD-aP^[35,36]^. One study showed that PTH levels correlated with the severity of pruritus in patients with HD^[37]^. This finding was not supported by other studies^[38–41]^. In a study on 105 patients with HD, there was no association between PTH or serum phosphorus and the presence or intensity of itch,^[13]^ but there was a positive correlation between CKD-aP and calcium serum concentration^[13]^. Several other studies found an association of CKD-aP with higher calcium, phosphorus, and PTH levels^[11,12,42,43]^. Other investigations recognized other factors such as longer periods of dialysis, men, *Kt/V*_urea_ (ratio representing fractional urea clearance) < 1.5, and lower serum levels of albumin, ferritin, and hemoglobin associated with pruritus^[11,34,44]^.

### Histamine release

The significance of histamine in CKD-aP is controversial^[45–48]^. Also, the increased number and spread of mast cells in the skin of patients with CKD-aP is debated^[49–53]^. Tryptase is another pruritogenic mediator released from the mast cells. Dugas-Breit et al^[54]^ revealed a significant correlation between serum levels of tryptase and itch severity. Similarly, serotonin serum levels were higher in patients with CKD-aP compared with the control group^[55]^. Also, Balaskas et al^[56]^ demonstrated that ondansetron, a 5HT_3_-receptor inhibitor, significantly reduced the severity of pruritus.

### Xerosis

There is a high co-occurrence of xerosis (dry skin) with pruritus in advanced CKD and HD patients with a prevalence of 50%–85%^[57,58]^. Dryness of the stratum corneum and increased skin pH, which happen in xerosis, are due to the sebaceous glands atrophy and the basement membrane thickening^[59]^. It is currently thought that xerosis is a risk factor associated with CKD-aP^[58]^. Also, xerosis affects the severity of itch^[7]^. A correlation between itch and reduced stratum corneum hydration was shown by Morton et al^[60]^ who also proved the efficacy of emollients in relieving pruritus. However, Yosipovitch et al^[61]^ did not find such a correlation between xerosis and itch. Also, Akhyani et al^[38]^ found no significant difference in xerosis prevalence between pruritic and nonpruritic HD patients. Given its low risk of side effects, emollient usage is recommended for patients with CKD-aP, and it has positive effects on the dryness of the skin, itch severity, and patient’s quality of life^[62–64]^.

### Immune system dysregulation

One theory posits that immune dysregulation and microinflammation in the skin and possibly systemic inflammation could participate in the pathogenesis of CKD-aP. Inflammatory markers seem to have higher levels in patients with CKD-aP. These markers include T-helper 1 cells, interleukin-2, interleukin-6, and C-reactive protein^[64,65]^. A potential role for interleukin-31 in the pathogenesis of CKD-aP can be considered. Interleukin-31, which can be produced by inflammatory cells but which acts on receptors on peripheral nerves is considered a mediator of prur itus itch^[66,67]^. While patients with CKD-aP had elevated serum levels of interleukin-31, a randomized, double-blind, placebo-controlled study in which the antipruritic effect of nemolizumab, a monoclonal antibody against interleukin-31 receptor, was assessed in HD patients with CKD-aP, a statistically significant reduction of pruritus severity scores was not observed^[68]^.

Other inflammatory markers might have a role. Several studies revealed that CKD-aP is associated with high white blood cell counts, low levels of albumin, and high levels of ferritin^[2,11,12,69]^.

After renal transplantation, patients on cyclosporine rarely suffered from itch as long as cyclosporine was administered, even when transplant function had a substantial loss^[70,71]^. In a case-control study, atorvastatin was associated with decreased frequencies of CKD-aP among HD patients, and authors considered atorvastatin to be a predictive factor associated with CKD-aP. They reported that the use of atorvastatin reduced the index of CKD-aP (95% CI: 0.256–0.954, odds ratio = 0.494). The uremic patients with atorvastatin experienced relief of pruritus 2.024 times more than uremic patients without atorvastatin (CI: 1.05–3.91) (*P* = 0.036)^[72]^. Also, Duque et al^[13]^ found that HD patients receiving statins reported CKD-aP significantly less than HD patients without statins. Statins act by inhibiting the presentations of intercellular adhesion molecule-I, monocyte chemotactic protein-I, and lymphocyte function-associated antigen-I on endothelial cells and leukocytes. The inhibitory effect of statins on the expression of major histocompatibility complex (MHC) class II is responsible for their immunomodulatory effect. Atorvastatin is a strong inhibitor of inducible MHC class II expression^[73]^. Atorvastatin interferes with T-helper cell (Th) regulation by obstruction of 3-hydroxy-3-methylglutaryl–coenzyme A (HMG-CoA) reductase which prevents the biosynthesis of cholesterol and isoprenoids. In addition, the phosphatidylinositol-3-kinase/Akt signal transduction pathway has an important role in different T-cell functions^[74,75]^. Moreover, statins reduce the migration of neutrophils and induce a prolonged reduction in neutrophil reactive oxygen species^[76]^. On the contrary, statins have been one of the medicines that can cause pruritus^[72,73]^. Inhibition of cholesterol biosynthesis leads to impaired barrier function and disrupts the distribution of the lipid in the skin. Differences in the pharmacokinetics of the drug in HD patients may result in the prominence of the anti-inflammatory effect of atorvastatin over barrier dysfunction in this population^[73,77]^. However, further investigation for immunomodulatory properties of statins in dermatology and CKD-aP is required.

### Opioid system dysregulation

There exists a noticeable overlap in the neurocircuitry pathway that transmits pain and itch sensations^[22]^. Compounds that block pain, opioids, are also recognized as pruritogen agents. Opioid receptors on the brain, peripheral nerves, keratinocytes, melano cytes, hair follicles, and immune cells play a significant role in itch^[78]^. Endogenous opioid overstimulation has been involved in the pathogenesis of cholestatic pruritus and CKD-aP^[79,80]^. It has been suggested that an imbalance of mu and kappa-opioid receptor activity occurs in CKD-aP. The concept is that mu-opioid receptor over-activation, together with kappa-opioid receptor blockade, leads to an increase in the sensation of itch^[81]^.

### Uremic neuropathy

Another etiopathogenesis of CKD-aP is dysfunction of the central or peripheral nervous system^[31]^. Defects in the peripheral sensory pathway, cortical hypersensitivity, low cortical inhibitory mechanisms, or a defective spinal cord inhibitory mechanism may result in chronic itch^[82]^. Peripheral sensorimotor neuropathy and dysautonomia are prevalent among HD patients, and this may play a role in their CKD-aP^[83]^. Also, HD patients with paresthesia and restless leg syndrome experience CKD-aP more at an increased rate^[24,83,84]^. B-type (brain) natriuretic peptide (BNP) is a neuropeptide that can activate pruriceptive neurons in mice. Elevated concentrations of serum BNP are detected in HD patients where weight is controlled poorly. Shimizu and colleagues found that serum levels of BNP were frequently elevated in HD patients. They implicated BNP as one of the possible causes of daytime CKD-aP^[85]^. Sorour and colleagues showed that neurotrophin-4 (NT-4) levels were elevated in the serum of uremic patients with CKD-aP and that there was a positive correlation between NT-4 levels and the severity of pruritus. These observations reveal that NT-4 may play a role in CKD-aP^[86]^.

### New hypothesis

Mas-related G protein–coupled receptors (MRGPR_s_), localized predominately to sensory neurons and mast cells, are important non-histramine receptors of itch. MRGPR_s_ are activated by many substances, including certain proteases, antimicrobial peptides, and substance P^[87]^. Among 10 human MRGPR_s_, MRGPRX1, X2, and X4, are characterized as itch receptors^[88,89]^. Drugs that target the MRGPRX4 receptor (EP547) are in clinical trials for itch. A phase I clinical study demonstrated a reasonable safety, tolerability, and pharmacokinetic profile. EP547 could be a potential therapy for chronic cholestatic and uremic pruritus. (ClinicalTrials.gov Identifier: NCT04510090)^[88]^.

## Treatment

**Figure 2.**
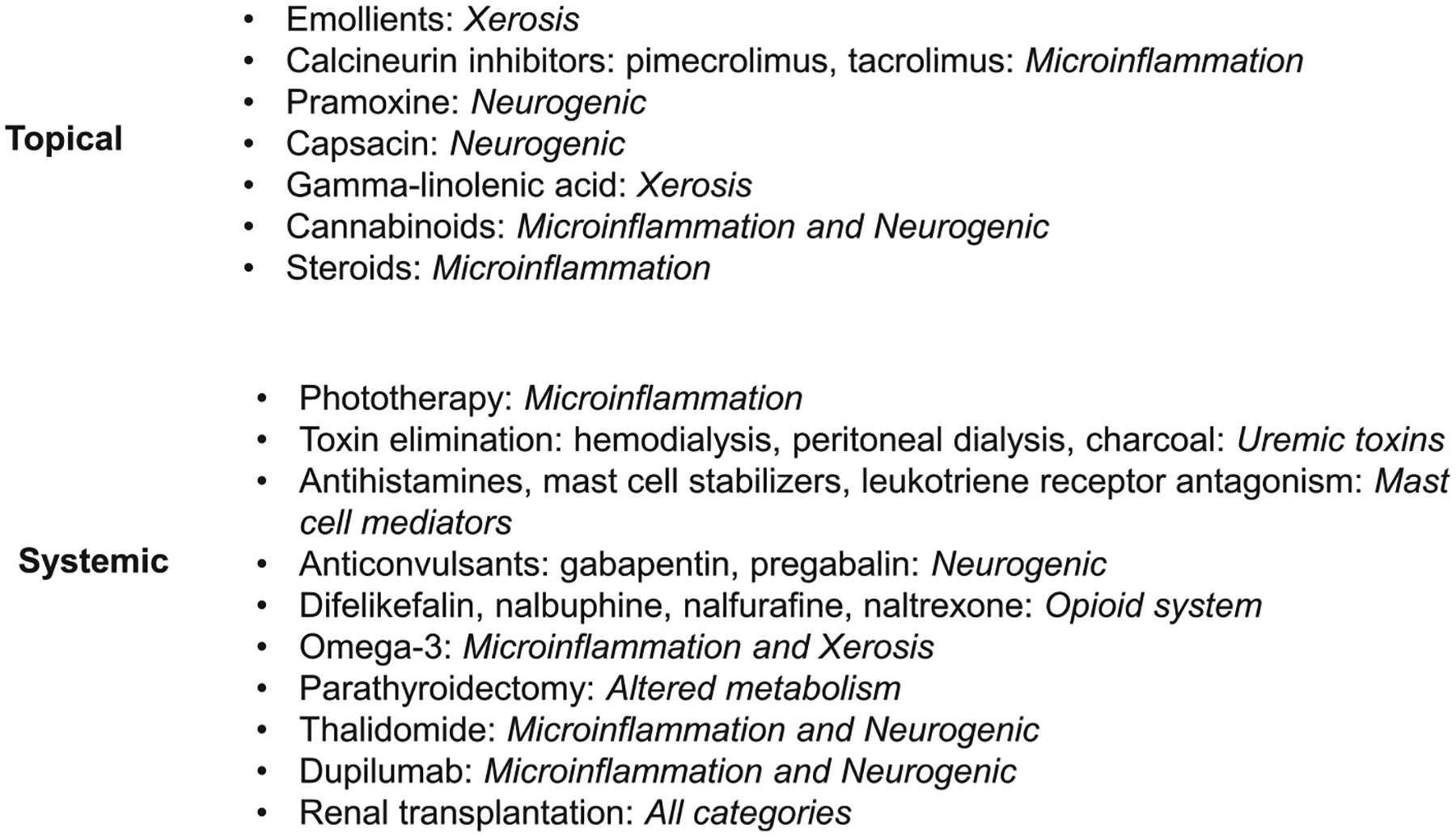
Current treatments for chronic kidney disease–associated pruritus and the potential mechanistic categories from Figure 1.

The etiopathogenesis of CKD-aP is not clearly understood. So, effective therapy for this bothersome condition remains a challenging task for nephrologists and dermatologists. Unfortunately, yet there is no treatment consensus for CKD-aP. Successful therapy can be achieved after a thorough history and physical examination to rule out other pruritic conditions in uremic patients and make a diagnosis of CKD-aP, considering the patient’s age, medications, comorbidities, and severity of their CKD-aP. Thus, CKD-aP management should be individualized case by case (Figure 2).

Most of the proposed treatments for CKD-aP are based on case series or small, non-controlled clinical trials. We found a few large, double-blind, randomized controlled trials assessing treatment in patients with CKD-aP^[90–92]^.

## Topical therapy

### Emollients

Xerosis is prevalent in patients with CKD-aP. The first step of CKD-aP management is providing adequate skin hydration. Different formulations of emollient creams have been studied before. In a randomized multicenter, double-blind clinical trial, 99 dialysis patients with moderate-to-severe CKD-aP were compared with glycerol 15% and paraffin 10% in an oil-in-water emulsion versus an oil-in-water emulsion alone. On day 7, treatment response was observed in 72 patients (73%) of the first group and 44 patients (44%) of the comparator. Also, the quality of life of the patients at the study end had a substantial improvement. The authors stated that successful management of uremic xerosis occurs when an appropriate emollient therapy is used^[93]^. Several other non-controlled trials have also revealed the positive effects of emollient compounds in patients with xerosis and CKD-aP^[60,62,94–96]^.

### Calcineurin inhibitors

The topical calcineurin inhibitors, tacrolimus and pimecrolimus, inhibit calcineurin and consequently prevent the transcription of IL-2 and other cytokines in T cells. The role of this mechanism in the pathophysiology and, thus, treatment of CKD-aP is not settled. In an uncontrolled study, Kuypers et al^[97]^ reported that topical tacrolimus, 0.1% and 0.3%, significantly reduced the severity of CKD-aP after 6 weeks. Pauli-Magnus et al^[98]^ reported that topical 0.03% tacrolimus ointment twice daily for 7 days, could dramatically reduce CKD-aP. On the other hand, a double-blind RCT revealed that 0.1% tacrolimus ointment was not more effective than placebo for reducing CKD-aP in HD patients^[99]^. Similarly, another double-blind RCT of HD patients using 1% pimecrolimus, showed a lack of significant antipruritic proficiency of topical pimecrolimus 1% as compared with placebo^[100]^.

### Pramoxine

Pramoxine, a topical anesthetic, has been proposed to modulate the itch sensation by interfering with the impulse transmission of sensory nerve fibers. In a randomized, double-blind, controlled comparative trial of 28 HD patients with moderate-to-severe CKD-aP receiving at least 3 months of hemodialysis, a topical lotion containing 1% pramoxine twice daily for 4 weeks was more effective than the control for decreasing the intensity of CKD-aP^[101]^.

### Capsaicin

Capsaicin, a transient receptor potential channel (TRPV1), is the active compound in chili peppers. Capsaicin inhibits pain and pruritus conduction by depleting neuropeptide substance P from sensory nerve terminals in the skin and peripheral nerves. Capsaicin has been useful for neuropathic itch disorders, including notalgia paraesthetica, postherpetic itch, and brachioradial pruritus^[102]^. In a double-blind, crossover RCT on 17 HD patients presented that capsaicin 0.025% cream was sig nificantly more effective than placebo, and the antipruritic effect remained 8 weeks after treatment. Aside from local burning, stinging, and erythema, there were no other significant side effects^[103]^. This finding was confirmed by Chu et al^[40]^.

### Gamma-linolenic acid

Gamma-linolenic acid is an essential fatty acid that may modulate the function of T lymphocytes and lymphokines. Chen and colleagues performed a prospective, randomized, double-blind, placebo-controlled, crossover study of 16 dialysis patients. They reported that a cream containing 2.2% gamma-linolenic acid applied on the pruritic sites 3 times a day for 2 weeks, and then the reverse treatment following a 2-week washout period, was more effective than the control cream for alleviating CKD-aP. They thus claim that gamma-linolenic acid is a useful adjuvant in the management of refractory CKD-aP^[104]^, although given the small study size, it is reasonable to maintain the possibility of an emollient effect of the active compound.

### Cannabinoids

Cannabinoids, a derivative of cannabis, are chemical compounds that may bring relief to CKD-aP. The anti-inflammatory and anti-nociceptive properties of endocannabinoids, through their effect on mast cells, histamine, and cytokines may contribute to their antipruritic effect^[105]^. In an uncontrolled study of 21 HD patients, a cream containing structured physiological lipids and endogenous cannabinoids (*N*-acetylethanolamine and *N*-palmitoylethanolamine) applied twice daily for 3 weeks led to a significant reduction in pruritus scores as measured by visual analog scale (VAS) with pruritus being eliminated in 38.1% of patients^[96]^. This study was referenced in a review of the use of marijuana and cannabinoids in CKD^[106]^. We are not aware of reports of the effect of oral or inhaled cannabinoids on CKD.

### Steroids

Topical steroids are effective for the treatment of inflamed skin. The skin of CKD patients is not inflamed, at least not in comparison to the skin of people with atopic dermatitis. It is thus not surprising that only 9% of physicians prescribe topical steroids as a first-line treatment for CKD-ap in HD patients^[2]^. We found no trials in which the efficacy of topical steroids has been evaluated.

## Systemic treatment

### Phototherapy

Ultraviolet (UV) therapy is used extensively in inflammatory skin diseases such as psoriasis and atopic dermatitis. The anti-inflammatory effect is not well-defined, but leads to an inhibition of Langerhans cells, and proinflammatory cytokines, apoptosis of mast cells, elevation of serum 25-hydroxyvitamin D3 levels, and the re-establishment of the skin barrier^[31]^. Many studies reported the usefulness of phototherapy for CKD-aP^[107–109]^. A meta-ana lysis in the 1990s reported the effectiveness of ultraviolet-B (UVB) radiation in improving CKD-aP, while ultraviolet-A (UVA) did not seem to be operative^[110]^. Unexpectedly, narrowband (NB)-UVB phototherapy did not show a significant effect regarding pruritus intensity for refractory CKD-aP compared with a control group^[109]^. The limited accessibility and numerous sessions of irradiation have limited the use of this approach in the management of CKD-aP^[7,31]^.

### Toxin elimination

There are conflicting results about the relationship between *Kt/V* values and the presence of CKD-aP in HD. Hiroshige et al^[111]^ demonstrated that higher dialysis efficacy with good nutritional condition decreases the prevalence and intensity of CKD-aP and *Kt/V* ≥ 1.5 and administration of a high-flux dialyzer may improve CKD-aP^[112]^. Conversely, the longer one is on dialysis, skin dryness, and higher *Kt/V* positively correlate with the intensity of CKD-aP^[13]^.

The effect of dialysis modification on CKD-aP has been assessed. The findings from 2 RCTs revealed that high-flux hemodialysis had a better effect in alleviating the CKD-aP as compared with low-flux hemodialysis and hemodialysis filtration^[113,114]^. In addition, in a single-center retrospective study on 144 HD patients, results showed that after 24 months of treatment, combined regular HD, hemodiafiltration, and hemo perfusion were more effective than combined HD and hemoperfusion^[115]^. In a randomized, prospective, double-blind study comparing the efficacy of high permeability hemodialysis (HPHD) against conventional hemodialysis (CHD) in 116 HD patients with CKD-aP, HPHD was thought to relieve CKD-aP through the clearance of accumulated mid and macro molecule (β2-microglobulin) toxins and parathyroid hormone^[116]^.

Binding agents, including activated carbon and cholestyramine, might act to remove putative pruritogens from the intestinal lumen^[117]^. The impact of oral charcoal on generalized CKD-aP in 11 stable patients undergoing maintenance hemodialysis was compared with that of placebo in a controlled, double-blind, crossover study. Charcoal, 6 g daily, was used for 8 weeks. CKD-aP intensity significantly improved compared with the placebo. Symptomatic relief and objective improvement of cutaneous lesions were observed, with no significant side effects^[118]^. Similar effectiveness for cholestyramine was achieved for CKD-aP alleviation in a double-blind, placebo-controlled trial on 10 HD patients^[119]^.

### Antihistamines and mast-cell stabilizers

Antihistamines are often used to treat chronic pruritus. According to a DOPPS survey, 57% of non-dermatologist medical directors prescribed antihistamines as an “appropriate” therapy for CKD-aP^[2]^. But literature review highlighted poor or moderate effects of histamine-receptor antagonists in CKD-aP^[95,117,120,121^]. Moreover, Combs and colleagues suggested in a review that the pruritus relief perceived after antihistamine administration may be due to a sedative mechanism, instead of an anti pruritic effect. The potential for over-sedation, particularly in the elderly, limits the use of histamine receptor antagonists (such as diphenhydramine, hydroxyzine, loratadine, or cetirizine) for the treatment of CKD-aP^[95]^.

Alternatively, some current studies have evidenced that the consumption of mast-cell stabilizers (such as cromolyn sodium, zinc sulfate, and ketotifen), which prevent histamine release, brings reasonable antipruritic effects in the CKD-aP treatment^[3]^. Studies showed that ketotifen and cromolyn sodium have been effective in reducing pruritus severity^[48,122]^. Also, cromolyn sodium 4% topical cream twice a day for 4 weeks could decrease the pruritus intensity compared with the placebo^[123]^.

Mahmudpour et al^[124]^ revealed in a double-blind RCT of 80 CKD-aP HD patients, those who received montelukast (leukotriene receptor blocker) for 30 days experienced less intensity in pruritus compared with the placebo.

### Anticonvulsants

Gabapentin and pregabalin are anticonvulsant drugs, an analog of the neurotransmitter gamma-aminobutyric acid (GABA), that are used in some types of seizures and neuropathic pain. Numerous studies revealed the effects of gabapentin and pregabalin on CKD-aP^[84,122,125–131]^. The assumed mechanism is binding to α2δ subunits of voltage-sensitive calcium channels and diminishing the influx of calcium into nerves^[132]^. No significant difference was found between gabapentin efficacy and ketotifen or pregabalin regarding CKD-aP treatment^[132]^. Pregabalin had fewer side effects and better tolerability than gabapentin^[133,134]^, but in a comparative cross-sectional study of 90 HD patients, sedation, nausea, and blurred vision were more frequent in patients who took pregabalin compared with those with gabapentin^[135]^.

### Opioid-receptor mediators

As mentioned above, dysregulation of mu and kappa receptors is linked to pruritus. Kappa agonism and mu antagonism can alleviate pruritus. The treatment of CKD-aP based on these mechanisms has been reported^[136]^.

Difelikefalin is a peripheral highly selective kappa-opioid receptor agonist for treating adult HD patients with moderate-to-severe CKD-aP. This drug may not cross the brain-blood barrier, limiting the central nervous system’s adverse effects otherwise associated with opioids. The potential for abuse is further limited by a lack of activity on mu receptors^[136]^. In a randomized, double-blind controlled trial of 174 HD with moderate-to-severe CKD-aP, patients received difelikefalin intravenously (0.5, 1.0, or 1.5 μg/kg) or placebo 3 times a week after each session of HD for 8 weeks. A significant reduction in CKD-aP intensity scores and improvement over placebo in Skindex-10, 5-D itch, and sleep disturbance scores occurred. About 80% of HD patients with difelikefalin reported side effects of diarrhea, dizziness, nausea, somnolence, and falls versus 42% of HD patients with a placebo^[137]^. Similarly, in another double-blind, placebo-controlled, phase 3 trial, HD patients with moderate-to-severe CKD-aP receive either 0.5 μg/kg of body weight intravenous difelikefalin or a placebo trice weekly for 12 weeks. 49.1% of patients described at least 3 points of pruritus severity (measured by the WI-NRS) improvement versus 27.9% of patients without treatment. Mild to moderate diarrhea, dizziness, and vomiting were the most common adverse events and subsided^[92]^. The Food and Drug Administration (FDA) approved difelikefalin in August 2021, as the first drug for the treatment of adult HD patients with CKD-aP. The European Medicines Agency (EMA) approved the difelikefalin in April 2022. The drug is administered as an intravenous injection with a recommended dose of 0.5 μg/kg body weight at the end of the hemodialysis session^[138]^. In a phase 2 randomized clinical trial on 247 HD patients with CKD-aP dife likefalin (0.5 or 1.0 μg/kg) for 8 weeks meaningfully reduced pruritus, as measured via the Numerical Rating Scale (NRS) score without identifying safety concerns^[139]^. Systematic reviews further support the efficacy, safety profile, and improved QOL of difelikefalin, such that this drug can be considered a primary treatment for pruritus in patients with CKD-aP. The long-term continued benefit remains to be determined^[140,141]^. A phase 2, a double-blind, randomized, placebo-controlled dose-finding study of oral difelikefalin (0.25, 0.5, or 1.0 mg) or placebo once daily for 12 weeks in 269 non-dialysis–dependent CKD (stage 3–5) and HD patients with moderate-to-severe pruritus was performed. Difelikefalin, 1.0 mg, significantly reduced mean WI-NRS scores weekly compared with the placebo at week 12, with numerical reductions detected with difelikefalin 0.25 and 0.5 mg. In the 12th week, 38.6% of cases with difelikefalin 1.0 mg experienced complete response (WI-NRS 0–1) versus 14.4% of cases with placebo. Difelikefalin also led to an improvement of up to nearly 20% in itch-related quality-of-life scores. Oral difelikefalin thus significantly decreased the intensity of itch in stage 3–5 CKD patients with moderate-to-severe pruritus^[142]^.

Another selective small molecule kappa-opioid receptor agonist, nalfurafine, was introduced as a potentially effective treatment for CKD-aP^[143,144]^. A phase 3 randomized, double-blind, placebo-controlled study on 337 HD patients with severe CKD-aP received oral nalfurafine (5 or 2.5 μg/d) or a matching placebo for 2 weeks, adjunctive to their existing antipruritic treatment(s). At the end of the study, 29% of patients receiving nalfurafine 2.5 μg/d, 32% of patients receiving nalfurafine 5, and 17% of patients receiving placebo experienced a significant improvement with more than 50% reduction in VAS scores^[90]^. In a pooled analysis of 2 multicenter, randomized, double-blind, placebo-controlled studies, CKD-aP patients received post dialysis intravenous treatment with either nalfurafine fine (n = 86) or placebo (n = 58) for 2–4 weeks. Statistically significant reductions in the mean of worst itching, intensity, excoriations, and sleep disturbances were found among patients with nalfurafine compared with placebo^[145]^.

A few studies evaluated the effect of the oral mu-receptor antagonist naltrexone in CKD-aP. Naltrexone 50 mg/d for 7 days could significantly reduce the CKD-aP in a group of 15 HD patients^[80]^. Conversely, in another study, naltrexone was not effective in CKD-aP. No difference was found between 26 HD patients with CKD-aP receiving naltrexone 50 mg/d and 26 HD patients with CKD-aP receiving loratadine 10 mg/d after a washout of 48 hours treated for 2 weeks^[146]^. A treatment algorithm for managing uremic pruritus in CKD-aP patients notes that naltrexone can be used off-label as a possible second-line therapy if not contraindicated^[147]^.

Nalbuphine is a mixed kappa-agonist/mu-antagonist. In an open-label, multiple escalating dose study, nalbuphine extended-release tablets up to 240 mg bid were found safe in HD patients without dose adjustment and 60 mg and 120 mg twice daily over 2 weeks, improved CKD-aP in 15 HD patients. The results were not significant, perhaps due to the small sample size^[148]^. In a multicenter, randomized, double-blind, placebo-controlled trial, 373 HD patients received nalbuphine extended-release tablets (60 or 120 mg) or placebo for 8 weeks. There was a significant reduction in itch intensity score and improved sleep disturbances in the patients receiving 120 mg in comparison to the placebo group^[91]^.

### Omega-3

In a systematic review, Panahi et al^[149]^ found multiple health benefits of omega-3 fatty acids in CKD and recommended that HD patients with CKD-aP take omega-3 as side effects were limited combined with the potential for effectiveness. A prospective cohort study of 27 HD patients with CKD-aP, who consumed 1000 mg fish oil per day for 3 months, determined that omega-3 polyunsaturated fatty acid significantly improved skin hydration on both the face and arms, as well as inflammation and disease-related consequent symptoms of pruritus^[150]^. These findings were supported by a meta-analysis, and the authors suggested 0.9–4.36 g of O3FA daily as a supplement daily^[151]^.

### Parathyroidectomy

In patients with ESRD and secondary hyperparathyroidism, parathyroidectomy can be effective in improving CKD-aP^[35,36,152^]. Parathyroidectomy is restricted to CKD-aP patients with concurrent hyperparathyroidism^[63]^. It is not known if PTH is a mediator of pruritus.

### Thalidomide

Thalidomide is known for its sedative, immunomodulatory, neuromodulatory, and antiangiogenic characteristics^[153]^. In a double-blind crossover RCT of 29 HD patients with severe CKD-aP, it was found that 55.6% of patients receiving thalidomide had reduced intensity of pruritus compared with 13.3% of patients receiving placebo. Potential side effects, such as teratogenicity, peripheral neuropathy, sedation, and constipation, should be noted before administration^[154]^.

### Biologics

Recent studies have reported that dupilumab, a human monoclonal antibody that blocks interleukin 4 and 13, may have be of benefit in CKD-aP^[155,156]^. Increased expression of interleukin-4 and/or interleukin-13 in uremic patients, and blocking of the signaling of interleukin-4 and/or interleukin-13 with dupilumab on sensory neurons are the assumed mechanisms^[155]^.

### Other therapeutic considerations

We recognize that in case reports or small unconfirmed studies, and without a clear mechanistic basis, additional therapeutics have been suggested^[9]^. These include, but are not limited to, turmeric, aprepitant, mirtazapine, and parenteral lidocaine, and are not discussed further.

### Renal transplantation

Panuccio et al^[157]^ showed that the prevalence of pruritus is reduced after renal transplantation (32% in renal transplant patients vs. 62% in HD patients) but remained considerably higher than in healthy subjects (11%) and had negative effects on the quality of life among these patients. In a single-center cohort of 132 patients who underwent renal transplantation between 1976 and 2014, only 66 patients remained in the study. The mean duration of time since renal transplantation was 10.17 ± 0.78 years. The authors showed that the prevalence and intensity of pruritus before transplantation were higher than after transplantation^[158]^.

In an unrelated study of 197 renal transplant recipients, the itch was present in 38.6% of the HD patients and ceased completely in 73.7% of patients after transplantation, while in 23.7%, the intensity of itch was lowered and only 2.6% of patients did not report any difference. However, 21.3% of subjects reported pruritus after renal transplantation, and 52.4% of patients’ itch developed after transplantation. The authors indicated that new-onset pruritus after renal transplantation demands attention^[159]^. The mechanism of new-onset itch may be a result of cutaneous drug reaction, persistent hyperparathyroid ism, or decreased graft function^[160]^.

## Conclusions

CKD-aP is a prevalent and often underappreciated challenge for HD patients. A better understanding of the underlying mechanisms of pruritus has the potential to provide additional treatment options for patients who suffer from CKD-aP. There is no treatment of choice. Difelikefalin is the only drug approved in the US or Europe for moderate-to-severe CKD-aP. Orally administered drugs that can benefit some patients include gabapentin, pregabalin, nalfurafine, and nalbuphine. Early results targeting MRGPRX4 with EP547 are worth expanding. We wonder if the many pathophysiologic mechanisms under consideration combined with the limited effectiveness of current therapeutics mask a potential simplicity that could underlie CKD-aP.
